# Prediction of cardiovascular risk in patients with chronic obstructive pulmonary disease: a study of the National Health and Nutrition Examination Survey database

**DOI:** 10.1186/s12872-021-02225-w

**Published:** 2021-09-01

**Authors:** Yun Shi, Jing Zhang, Yingshuo Huang

**Affiliations:** 1 Department of Geriatrics, Beijing Friendship Hospital, Capital Medical University, Beijing, 100050 People’s Republic of China; 2 Research Ward, Beijing Friendship Hospital, Capital Medical University, No. 95, Yong’an Road, Xicheng District, Beijing, 100050 People’s Republic of China

**Keywords:** Cardiovascular disease, Chronic obstructive pulmonary disease, Predictive model, NHANES database

## Abstract

**Background:**

Cardiovascular disease (CVD) is a common comorbidity associated with chronic obstructive pulmonary disease (COPD), but few studies have been conducted to identify CVD risk in COPD patients. This study was to develop a predictive model of CVD in COPD patients based on the National Health and Nutrition Examination Survey (NHANES) database.

**Methods:**

A total of 3,226 COPD patients were retrieved from NHANES 2007–2012, dividing into the training (n = 2351) and testing (n = 895) sets. The prediction models were conducted using the multivariable logistic regression and random forest analyses, respectively. Receiver operating characteristic (ROC) curves, area under the curves (AUC) and internal validation were used to assess the predictive performance of models.

**Results:**

The logistic regression model for predicting the risk of CVD was developed regarding age, gender, body mass index (BMI), high-density lipoprotein (HDL), glycosylated hemoglobin (HbA1c), family history of heart disease, and stayed overnight in the hospital due to illness last year, which the AUC of the internal validation was 0.741. According to the random forest analysis, the important variables-associated with CVD risk were screened including smoking (NNAL and cotinine), HbA1c, HDL, age, gender, diastolic blood pressure, poverty income ratio, BMI, systolic blood pressure, and sedentary activity per day. The AUC of the internal validation was 0.984, indicating the random forest model for predicting the CVD risk in COPD cases was superior to the logistic regression model.

**Conclusion:**

The random forest model performed better predictive effectiveness for the cardiovascular risk among COPD patients, which may be useful for clinicians to guide the clinical practice.

## Background

Chronic obstructive pulmonary disease (COPD), a common preventable and treatable disease, is characterized by persistent airflow limitation, COPD is frequently associated with increased airway and lung inflammatory responses to noxious particles or gases, [1], and will be the third leading cause of deaths all over the world by 2030 [2]. It occurs in approximately 10% of adults older than 40 years [3]. Disease management is complicated by the presence of comorbidities, which are an important component of COPD [4, 5]. Furthermore, significant comorbidities may have an impact on the incidence and mortality of COPD [6]. The common comorbidities of COPD include cardiovascular disease (CVD), skeletal muscle wasting, and stroke [7–9]. Of which, CVD is widely considered to have the greatest impact on COPD patients and is associated with disease progression, clinical outcomes, and mortality [10, 11].

Several mechanisms have been proposed to explain the link between COPD and increased risk of CVD [12–14]. COPD patients are at greater risk of CVD compared with age-matched and sex-matched individuals without COPD [7, 15]. In addition, COPD patients with CVD report more dyspnea, poorer quality of life, more frequent hospitalizations, and higher mortality than those with COPD alone [16]. CVD and COPD have similar risk factors, which are frequently coexist, such as aging, history of cigarette smoking, and a sedentary lifestyle [17–19]. However, the risk of CVD in most COPD patients has not yet been identified [20]. Predicting CVD risk is of great significance for disease management of COPD, including timely intervention and rational drug use.

In the current study, we assessed the variables of CVD risk in patients with COPD, and developed models that using multivariable logistic regression and random forest analysis to predict the risk of CVD in COPD patients. Also, the performance of these models was investigated with the internal validation.

## Methods

### Study design and data source

The data were extracted from NHANES (2007–2012) database [21], a cross-sectional survey of the U.S. civilian. Information was collected via household interviews and standardized physical examinations in specially equipped mobile examination centers. A total of 3226 COPD adults aged 40 to 79 years were enrolled in this study, dividing into the training (n = 2351) and testing (n = 895) sets. The approval from the Institutional Review Board of Beijing Friendship Hospital, Capital Medical University was not required because the data accessed from NHANES were freely available.

### Measurement of diseases

COPD was confirmed based on the Medical Conditions Questionnaire (MCQ), including “Ever told you had emphysema” (MCQ160G) and “Ever told you had chronic bronchitis” (MCQ160K). The participants would be diagnosed as COPD if one of the two questions were answered yes. In addition, if the subjects underwent two pulmonary function measurements, the mean value of baseline 1st test spirometry-forced expiratory volume in the first 1.0s (SPXNFEV1) and bronchodilator 2nd test spirometry-forced expiratory volume in the first 1.0s (SPXBFEV1) was taken as FEV1, and the mean value of baseline 1st test spirometry-forced vital capacity (SPXNFVC) and bronchodilator 2nd test spirometry- forced vital capacity (SPXBFVC) was taken as FVC. If only one pulmonary function measurement was performed, the ratios of FEV1 (measured by SPXNFEV1) and FVC (measured by SPXNFVC) were calculated. FEV1 was predicted by gender, age and height in the whole population according to different races. The participants would be diagnosed as COPD when the actual FEV1 was less than 80% of the predicted value, and the actual FEV1/FVC was less than 70% of the predicted value.

CVD were determined respectively according to the questions “Ever told you had angina or heart failure” (MCQ160B), “Ever told you had heart attack” (MCQ160E), and “Has a doctor or other health professional ever told you that you had coronary heart disease” (MCQ160C). The individuals would be diagnosed as CVD if one of the three questions were answered yes.

### Determinants

Variables were extracted from the NHANES database containing demographic information, health-related characteristics, and healthcare-related characteristics. Demographic information was as follows: age, gender, ethnicity, education, and poverty income ratio. Health-related characteristics included general health, general health compared with last year, body mass index (BMI), smoking, anyone smoking inside the home, total smokers inside the home, cotinine (nicotine metabolites), 4-(methylnitrosamino)-1-(3-pyridyl)-1-butanol (NNAL, nicotine metabolites), sedentary activity per day, systolic blood pressure, diastolic blood pressure, high-density lipoprotein (HDL), glycosylated hemoglobin (HbA1c), family history of heart disease, and asthma. Healthcare-related characteristics covered healthcare place type, number of times received healthcare over past year, stayed overnight in the hospital due to illness last year, and number of overnight stays in the hospital due to illness.

### Statistical analysis

All statistical analyses were performed using SAS software (version 9.4) and scikit-learn (version 0.23.1). Scikit-learn (version 0.23.1) is a Python-based open-source machine learning library and used to implement the random forest model. The quantitative data were respectively described as the median and quartile [M (Q1, Q3)] through Mann–Whitney *U* test. N (%) was used to express the categorical data using ${\upchi }^{2}$ test. Data distribution were adjusted with the sample weight of the Mobile Examination Center (MEC) to deal with the oversample of the data itself. Since the data missing ratio was less than 10%, the mean or mode of the weighted sample was directly used to filling. All COPD cases were divided into the training and testing sets. Variables with significant differences were into the multivariable logistic regression model using the training set, and then internal validation with the testing set were to assess the predictive effectiveness of the CVD risk among COPD patients. Similarly, a random forest model for the risk prediction of CVD was carried out using the training set, and then the testing set was used to internally validate the model performance. *P* < 0.05 was considered statistically significant.

## Results

### Baseline characteristics

A total of 3226 COPD cases were included form the NHANES (2007–2012). Of which, 2351 COPD patients identified through the spirometry data were included in the training set, while another 895 COPD patients identified by questionnaire data were included in the testing set. The characteristics of the training set were shown in Table 1. There were 428 patients in COPD & CVD group and 1923 cases in COPD group, respectively. Compared with COPD group, the patient’s age (Z = − 2097.465, *P* < 0.001), male ratio (χ^2^ = 13.470, *P* < 0.001), education (χ^2^ = 8.905, *P* = 0.012), and poverty income ratio (Z = − 648.776, *P* < 0.001) were higher in COPD & CVD group. The overall health status of patients in COPD group was better than that in COPD & CVD group (χ^2^ = 57.185, *P* < 0.001), while the BMI (Z = 1084.647, *P* < 0.001), smoking ratio (χ^2^ = 16.890, *P* < 0.001), sedentary activity per day (Z = − 894.344, *P* < 0.001), systolic blood pressure (Z = 34.157, *P* < 0.001), HDL (Z = − 1256.720, *P* < 0.001), HbA1c (Z = 1539.918, *P* < 0.001), and family history of heart disease ratio (χ^2^ = 20.298, *P* < 0.001) were lower in COPD group than those in COPD & CVD group. The number of times received healthcare over past year (χ^2^ = 53.250, *P* < 0.001) and number of overnight stays in the hospital due to illness (χ^2^ = 501.298, *P* < 0.001) were less in COPD group than that in COPD & CVD group.

**Table 1:** Univariable analysis between COPD & CVD and COPD groups

Characteristics^a^	Group^b^		Statistics	*P*
	CODP & CVD (n = 428)	Only COPD (n = 1,923)		
**Basic Characteristics**				
Age (year), M (Q1, Q3)	68.00 (57.00, 74.00)	57.00 (48.00, 66.00)	Z = − 2097.465	< 0.001
Gender, n (%)			χ^2^ = 13.470	< 0.001
Male	265 (59.54)	911 (46.09)		
Female	163 (40.46)	1012 (53.91)		
Ethnicity, n (%)			χ^2^ = 4.144	0.247
Hispanic	91 (8.73)	478 (10.58)		
White	228 (74.11)	803 (69.02)		
Black	86 (10.38)	473 (12.66)		
Others	23 (6.78)	169 (7.74)		
Education, n (%)			χ^2^ = 8.905	0.012
Less than High School	189 (31.56)	736 (26.92)		
High school	106 (30.91)	452 (24.71)		
Any college	131 (37.53)	732 (48.38)		
PIR, M (Q1, Q3)	1.85 (1.07, 3.93)	2.64 (1.26, 4.54)	Z = − 648.776	< 0.001
**Health related characteristics**				
General health, n (%)			χ^2^ = 57.185	< 0.001
Very good or excellent	38 (13.74)	461 (31.75)		
Good	109 (30.38)	693 (36.92)		
Fair or poor	281 (55.88)	768 (31.33)		
General health compared with last year, n (%)			χ^2^ = 11.380	0.003
Better	79 (19.38)	347 (17.17)		
Worse	234 (55.56)	1235 (66.64)		
Same	114 (25.06)	341 (16.20)		
BMI (kg/m^2^), M (Q1, Q3)	30.10 (25.90, 30.10)	27.80 (23.90, 32.40)	Z = 1084.647	< 0.001
Smoke, n (%)			χ^2^ = 16.890	< 0.001
Yes	298 (71.88)	1071 (57.86)		
No	130 (28.12)	851 (42.14)		
Anyone smoke inside home, n (%)			χ^2^ = 1.435	0.231
Yes	111 (26.25)	418 (22.36)		
No	312 (73.75)	1494 (77.65)		
Total smokers inside home	1.00 (1.00, 2.00)	1.00 (1.00, 2.00)	Z = 170.919	< 0.001
Cotinine (ng/g urinary creatinine), M (Q1, Q3)	1.43 (0.20, 1467.86)	0.82 (0.22, 1169.49)	Z = 93.327	< 0.001
NNAL (10^–2^ ng/g urinary creatinine), M (Q1, Q3)	4.44 (0.52, 304.85)	2.21 (0.60, 227.80)	Z = 252.738	< 0.001
Sedentary activity per day (minutes), M (Q1, Q3)	360.00 (240.00, 480.00)	300.00 (180.00, 480.00)	Z = − 894.344	< 0.001
Systolic blood pressure (mmHg), M (Q1, Q3)	126.00 (112.00, 142.00)	124.00 (114.00, 138.00)	Z = 34.157	< 0.001
Diastolic blood pressure (mmHg), M (Q1, Q3)	66.00 (60.00, 74.00)	72.00 (64.00, 80.00)	Z = − 1431.932	< 0.001
HDL (mmol/L), M (Q1, Q3)	1.14 (0.96, 1.40)	1.29 (1.06, 1.60)	Z = − 1256.730	< 0.001
HbA1c, M (Q1, Q3)	5.90 (5.50, 6.60)	5.60 (5.30, 6.00)	Z = 1539.918	< 0.001
Family history of heart disease, n (%)			χ^2^ = 20.298	< 0.001
Yes	107 (31.04)	257 (14.54)		
No	291 (68.96)	1586 (85.47)		
Asthma, n (%)			χ^2^ = 5.175	0.023
Have	108 (27.09)	380 (19.61)		
Haven’t	319 (72.91)	1542 (80.39)		
**Healthcare Related characteristics**				
Healthcare place type, n (%)			χ^2^ = 7.518	0.057
Clinic or health center	82 (17.12)	431 (21.79)		
Doctor's office or HMO	305 (78.93)	1191 (73.12)		
Hospital	21 (3.68)	109 (4.13)		
Others	3 (0.26)	17 (0.96)		
Number of times received healthcare over past year, n (%)			χ^2^ = 77.193	< 0.001
0	11 (2.18)	204 (10.81)		
1	21 (5.21)	229 (12.32)		
2–3	75 (20.45)	485 (26.66)		
4–9	150 (35.05)	615 (33.18)		
10–12	81 (17.20)	177 (7.63)		
13 or more	90 (20.91)	211 (9.40)		
Stayed overnight in the hospital due to illness last year, n (%)			χ^2^ = 53.250	< 0.001
Yes	189 (42.35)	386 (16.92)		
No	239 (57.65)	1535 (83.08)		
Number of overnight stays in the hospital due to illness, n (%)	1.00 (1.00, 2.00)	1.00 (1.00, 2.00)	Z = 501.298	< 0.001

^a^PIR–poverty income ratio, BMI–body mass index, NNAL–4-(methylnitrosamino)-1-(3-pyridyl)-1-butanonol, HMO–health maintenance organizations

^b^Categories may not sum to the total due to missing data

### The characteristics of cases between COPD & CVD and COPD groups

**Fig. 1:**
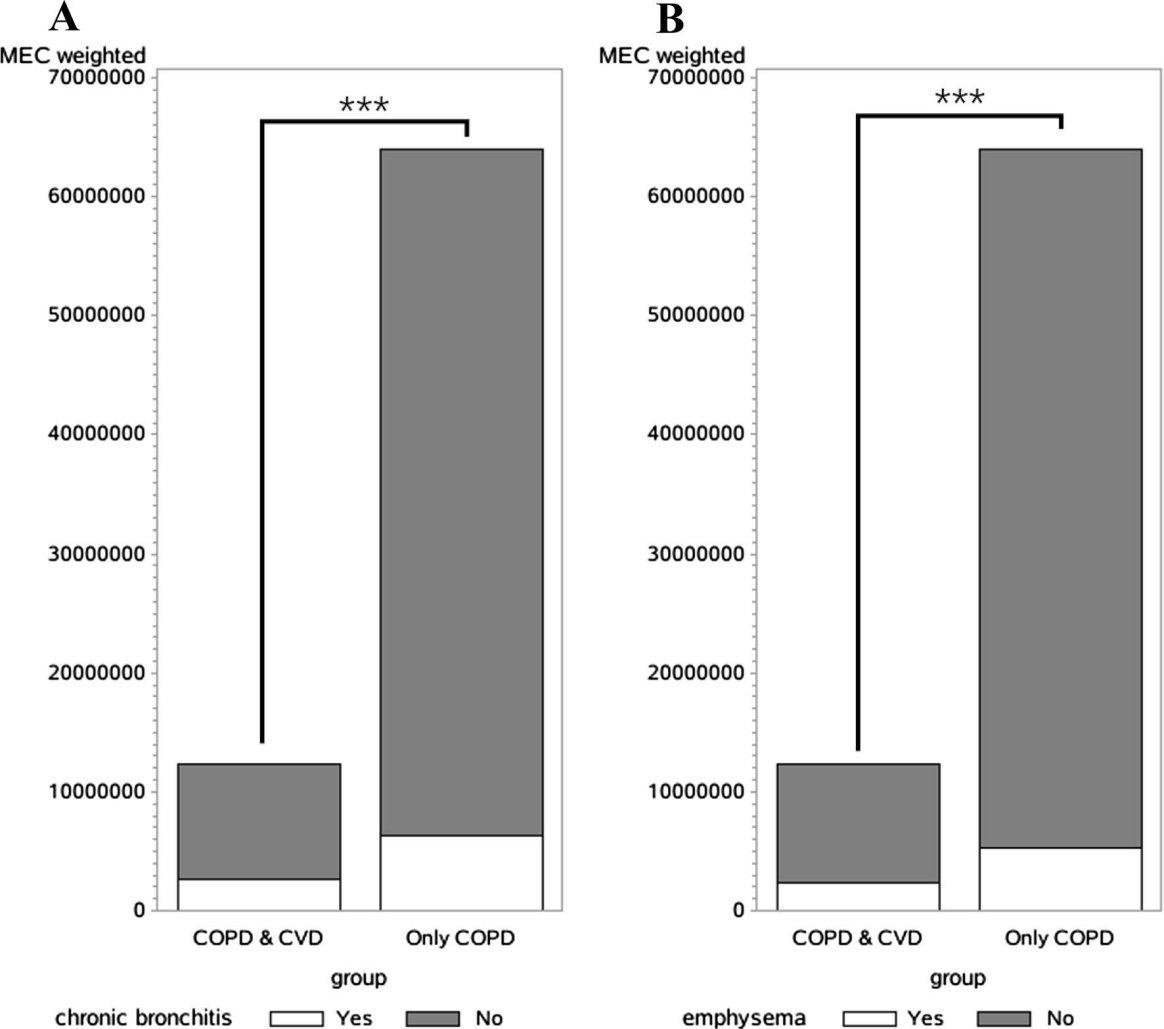
The proportion of patients with chronic bronchitis or emphysema in the COPD & CVD and COPD groups. **A** Patients with chronic bronchitis; **B** patients with emphysema

The proportion of patients with chronic bronchitis or emphysema was shown in Fig. 1. The results indicated that the COPD & CVD group patients had higher proportion of chronic bronchitis (19.45% vs 8.29%, χ^2^ = 16.689, *P* < 0.001) and emphysema (21.88% vs 9.96%, χ^2^ = 15.207, *P* < 0.001) than those in COPD group. In addition, there was no statistical difference in the distribution of angina, heart attack, heart failure, and coronary between males and females in the COPD & CVD group.

### The Logistic regression model for predicting CVD risk in COPD patients

The multivariate Logistic regression analysis was carried out to assess the determinants of CVD risk in COPD cases (Table 2). The results showed that age (OR 1.073, 95% CI 1.054 to 1.092), BMI (OR 1.025, 95% CI 1.003 to 1.048), HbA1c (OR 1.192, 95% CI 1.073 to 1.323), family history of heart disease (OR 2.665, 95% CI 1.79 to 3.967), and stayed overnight in the hospital due to illness last year (OR 2.314, 95% CI 1.543 to 3.551) were determinants for the risk of CVD among COPD patients. In addition, elevated HDL (OR 0.417, 95% CI 0.255 to 0.681) and females (OR 0.537, 95% CI 0.384 to 0.751) were associated with a reduced risk of CVD in COPD patients.

**Table 2:** Multivariable Logistic regression model for predicting CVD risk in COPD patients

Factors	β	t	*P*	OR (95%CI)
Age (year)	0.071	7.900	<0.001	1.073 (1.054, 1.092)
Gender				
Male	Ref			
Female	− 0.311	− 3.730	< 0.001	0.537 (0.384, 0.751)
Education				
Less than High School	Ref			
High school	0.267	2.020	0.049	1.551 (0.975, 2.469)
Any college	− 0.094	− 0.860	0.395	1.082 (0.735, 1.592)
PIR	− 0.033	− 0.570	0.570	0.967 (0.861, 1.087)
General health				
Very good or excellent	− 0.316	− 2.000	0.051	0.478 (0.306, 0.748)
Good	− 0.106	− 0.770	0.448	0.590 (0.412, 0.845)
Fair or poor	Ref			
General health compared with last year				
Better	0.134	0.870	0.388	1.117 (0.678, 1.839)
Worse	Ref			
Same	− 0.157	− 1.340	0.185	0.835 (0.583, 1.196)
BMI (kg/m^2^)	0.025	2.270	0.028	1.025 (1.003, 1.048)
Smoke				
Yes	0.121	1.180	0.242	1.274 (0.845, 1.922)
No	Ref			
Cotinine (ng/g urinary creatinine)	0.017	0.850	0.397	1.017 (0.978, 1.057)
NNAL (10^–2^ ng/g urinary creatinine)	0.780	0.130	0.900	2.182 (2.025, 2.451)
Sedentary activity per day (minutes)	0.000	0.290	0.774	1.000 (0.999, 1.001)
Systolic blood pressure (mm Hg)	− 0.009	− 1.690	0.097	0.991 (0.98, 1.002)
Diastolic blood pressure (mm Hg)	− 0.008	− 1.190	0.238	0.992 (0.979, 1.005)
HDL (mmol/L)	− 0.875	− 3.580	< 0.001	0.417 (0.255, 0.681)
HbA1c (%)	0.175	3.370	0.002	1.192 (1.073, 1.323)
Family history of heart disease				
Yes	0.490	4.950	< 0.001	2.665 (1.790, 3.967)
No	Ref			
Asthma				
Have	0.128	1.330	0.190	1.292 (0.877, 1.905)
Haven’t	Ref			
Number of times received healthcare				
Over past year				
0	Ref			
1	− 0.135	− 0.340	0.733	1.995 (0.662, 6.015)
2–3	0.211	1.280	0.207	2.819 (1.355, 5.867)
4–9	− 0.085	− 0.670	0.504	2.097 (1.007, 4.367)
10–12	0.440	1.690	0.097	3.545 (1.436, 8.753)
13 or more	0.393	1.850	0.071	3.38 (1.434, 7.967)
Stayed overnight in the hospital due to illness last year				
Yes	0.425	4.100	< 0.001	2.341 (1.543, 3.551)
No	Ref			

**Fig. 2:**
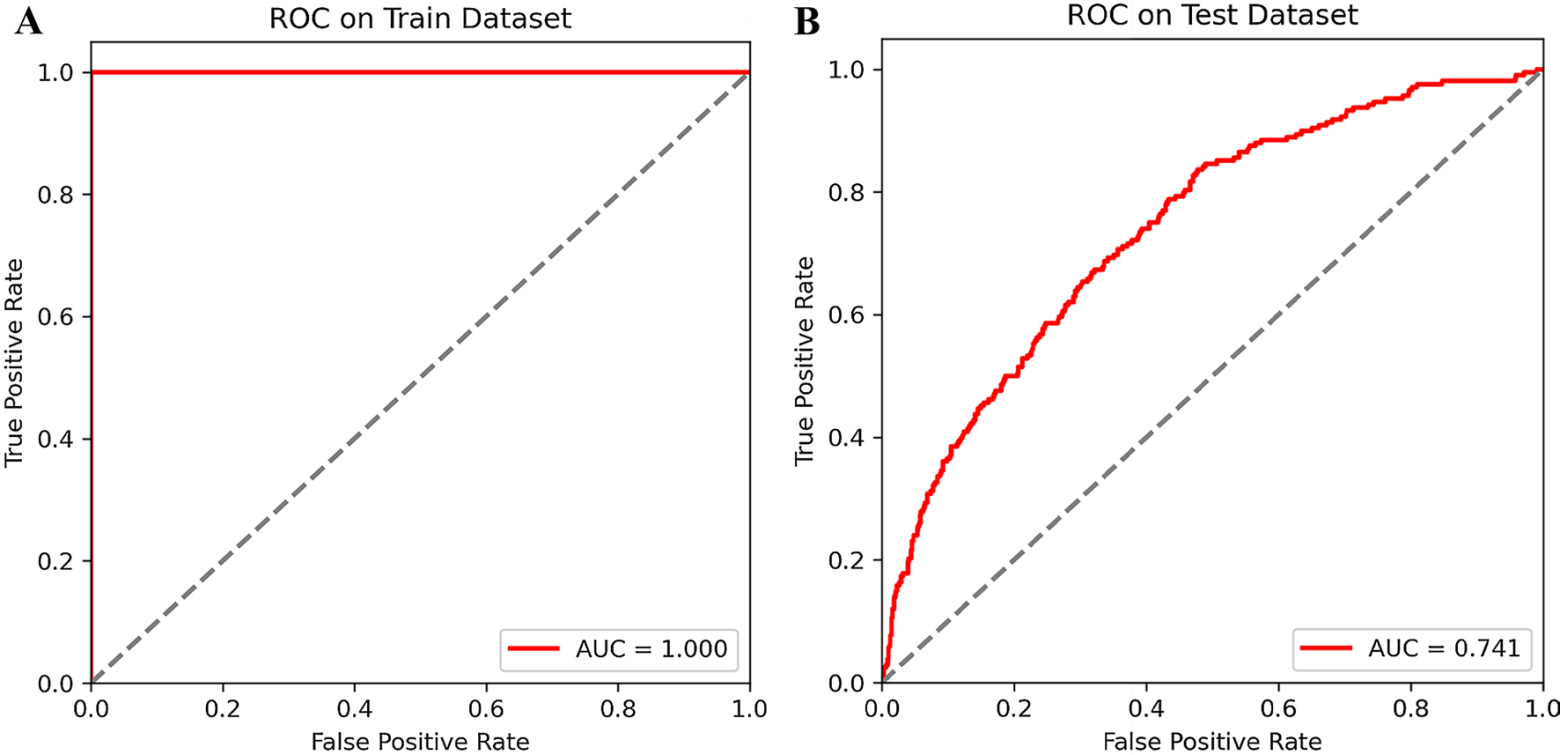
Receiver operating characteristic (ROC) curves and area under the curves (AUC) of Logistic regression model on the training set (**A**) and test set (**B**)

The Logistic regression model was established based on these determinants using the training set. The receiver operating characteristic (ROC) curves of the predictive model were plotted in Fig. 2. The area under curve (AUC) of the model was 1.000. The result of the internal validation with the test set showed that the AUC was 0.741, suggesting the model could be used to predict CVD risk in COPD patients.

### The random forest model for predicting CVD risk in COPD patients

**Fig. 3:**
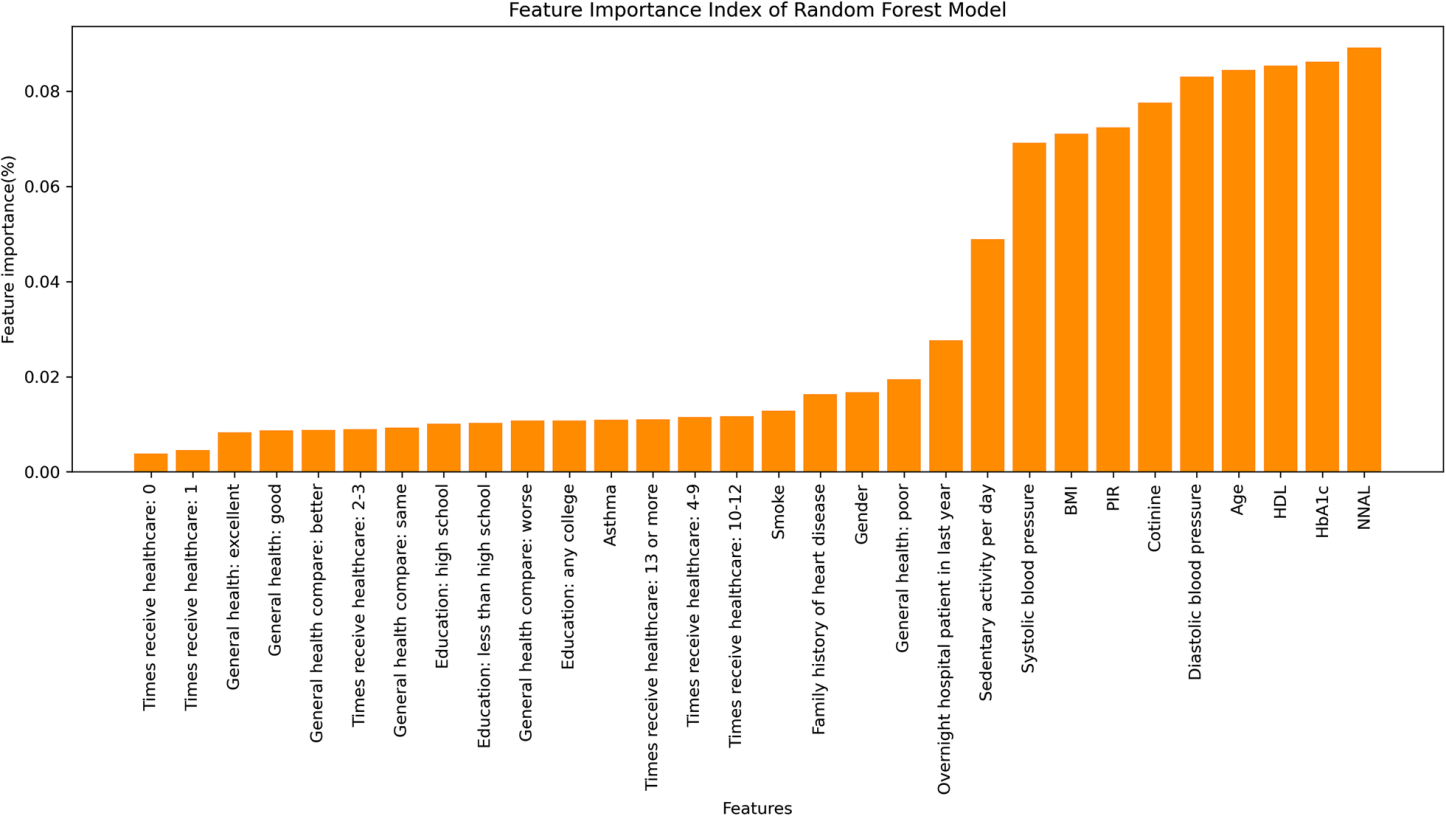
Importance of variables in the random forest model. NNAL-4-(methylnitrosamino)-1-(3-pyridyl)-1-butanol (nicotine metabolites), PIR-poverty income ratio

Totally 1024 decision trees were used in the random forest analysis, the maximum number of sampling features was 4, and the rest were set using default parameter. The important variables-associated with CVD risk were NNAL, HbA1c, HDL, age, gender, diastolic blood pressure, cotinine, poverty income ratio, BMI, systolic blood pressure, and sedentary activity per day. The importance of variables was listed in Fig. 3.

**Fig. 4:**
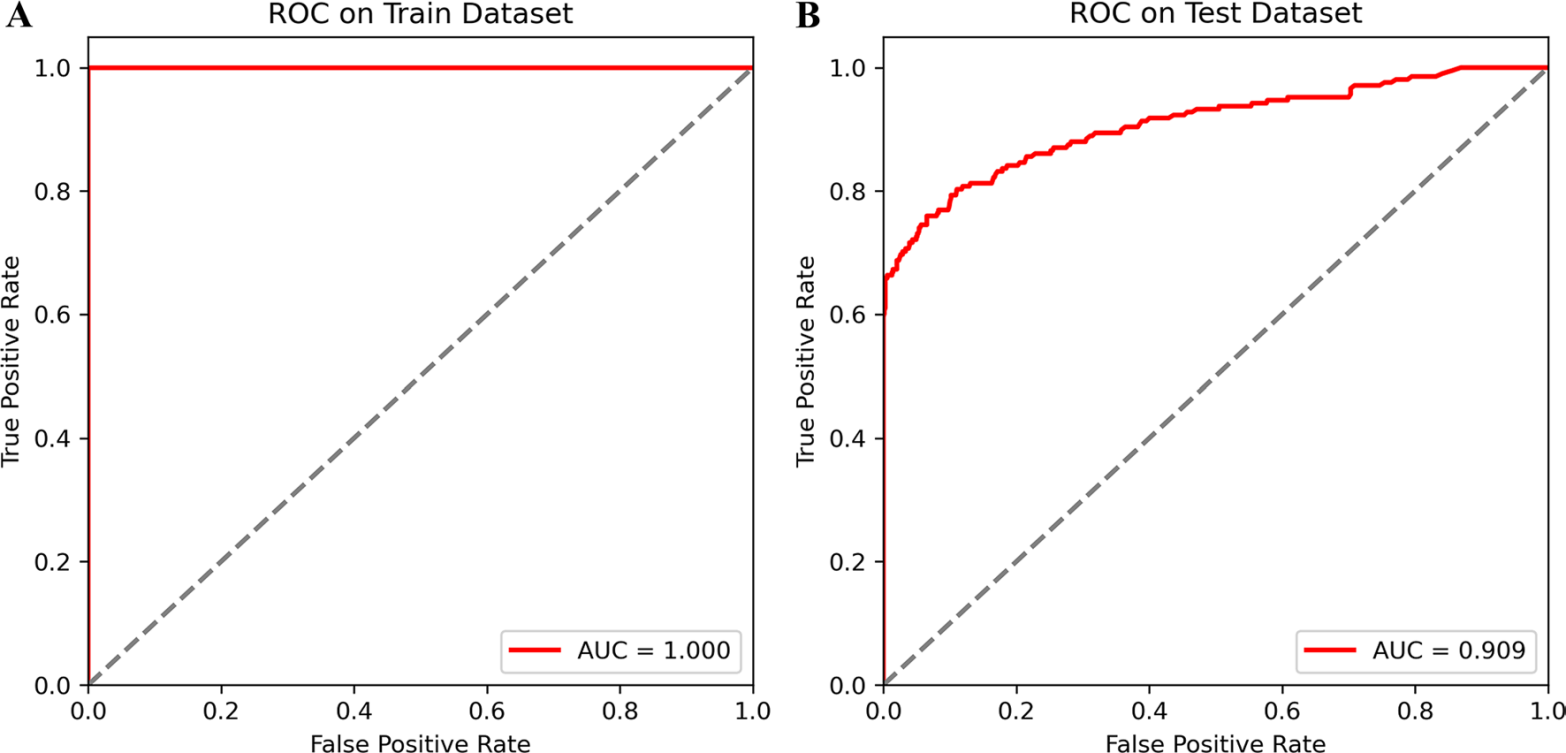
ROC curves and AUC of random forest model on the training set (**A**) and test set (**B**)

The ROC curves of the random forest model were displayed in Fig. 4. The AUCs of this model and the internal validation were 1.000 and 0.948, respectively. It was indicated that the random forest model was performed well predictive effectiveness in predicting CVD risk among COPD patients, which may be used to guide the clinical practice.

## Discussion

Two predictive models based on the NHANES database were carried out to identify CVD risk in COPD patients. The results showed that the AUC was 0.741 in the logistic regression model, and age, gender, BMI, HDL, HbA1c, family history of heart disease, and stayed overnight in the hospital due to illness last year were the determinants for the risk of CVD in COPD patients. The elevated HDL and females were associated with a reduced risk of CVD in COPD patients. In addition, the AUC of the random forest model was 0.948, and NNAL, HbA1c, HDL, age, gender, diastolic blood pressure, cotinine, poverty income ratio, BMI, systolic blood pressure, and sedentary activity per day were important variables-associated with CVD risk. The predictive efficiency of the random forest model was superior to the logistic regression model, indicating that the random forest model may perform better effectiveness in predicting the cardiovascular risk among cases with COPD.

Previous studies have reported a high incidence of COPD patients with CVD, leading to poor quality of life, dyspnea, low exercise tolerance and high risk of hospitalization [15, 22]. To reduce the risk of poor prognosis in COPD patients, it is necessary to effectively identify CVD risk. It was reported that multiple major risk factors-associated with CVD were found in COPD patients [23]. In our study, age, gender, BMI, HDL, HbA1c, were the important determinants of CVD risk in COPD patients in the logistic regression model. In the random forest model, metabolites associated with smoking (NNAL and cotinine), HbA1c, HDL, age, gender were the important variables of CVD risk in COPD patients. Variables such as age, gender, and HDL were important for CVD risk in COPD patients in both models. The study of Cazzola et al. indicated that patients > 35 years had higher odds ratio of simultaneous CVD and COPD [24]. Gunay et al. found that HDL level of COPD patients was significantly lower than that of healthy subjects [25]. Our results showed that higher age, males, and lower HDL level were associated with an increased risk of CVD in COPD patients.

To our best knowledge, the identification of cardiovascular risk in patients with COPD is currently unclear [20]. Few previous studies have developed a clinical predictive model that can be used to identify CVD risk in COPD patients. An early study showed that less than one-third of COPD patients were diagnosed with CVD by electrocardiographic images [26]. A simple, safe and effective method was needed to identify CVD risk in COPD patients. One recent study reported a model for predicting cardiovascular risk in patients with COPD, and their overall cardiovascular risk model C-statistic was 0.689 [27]. Our study provided two predictive models to identify CVD risk in COPD patients, especially the random forest model had a better predictive effect with an AUC of 0.948. The study of Adamson et al. indicated that cardiac troponin I concentrations were a specific and major biomarker of CVD risk in COPD patients [28]. In further studies, some important biomarkers can be included to improve the prediction effect of the model.

At present, the mechanism link between COPD and CVD is complex, multifactorial, and not yet fully understood [29]. Models that predicting CVD risk in COPD patients play an important role. The random forest model performed an excellent predictive effect and was simple on predicting CVD risk in COPD patients, which has the potential to be further applied to clinical practice. In future studies, more effective predictive model will be established to identify CVD risk among COPD patients. However, our study has some limitations. First, the diagnosis criteria of some COPD patients and all CVD patients relied on questionnaire data, which may affect the results of the model. However, in the random forest model, the model’s AUC had a small difference between the training set (COPD identified by spirometry data) and the test set (COPD identified through questionnaire data), indicating that the difference between COPD patients identified by questionnaire data and spirometry data was small. Second, an independent external validation study would be a more rigorous test and should be conducted. Third, the samples were mainly American, and there would be selection bias.

## Conclusion

This study provided two predictive models to identify CVD risk in COPD patients. The AUC was 0.741 in the logistic regression model, and age, gender, BMI, HDL, HbA1c, family history of heart disease, and stayed overnight in the hospital due to illness last year were the influencing factors for CVD in COPD patients. In the random forest model, the AUC was 0.948, and NNAL, HbA1c, HDL, age, gender, diastolic blood pressure, cotinine, poverty income ratio, BMI, systolic blood pressure, and sedentary activity per day were important variables-associated with CVD risk. The random forest model performed an excellent predictive effect on predicting CVD risk in COPD patients, which has the potential to be further applied to clinical practice.

## Abbreviations

CVD: Cardiovascular disease
COPD: Chronic obstructive pulmonary disease
ROC: Receiver operating characteristic
AUC: Area under the curves
BMI: Body mass index
HDL: High-density lipoprotein
HbA1c: Glycosylated hemoglobin
MCQ: Medical Conditions Questionnaire
FEV1: Forced expiratory volume in one second
FVC: Forced vital capacity

## Declarations

### Availability of data and materials

The datasets generated and/or analyzed during the current study are available in the NHANES, https://www.cdc.gov/nchs/nhanes/index.html.
